# Plant-Based Beverages as Good Sources of Free and Glycosidic Plant Sterols

**DOI:** 10.3390/nu10010021

**Published:** 2017-12-29

**Authors:** Anneleen I Decloedt, Anita Van Landschoot, Hellen Watson, Dana Vanderputten, Lynn Vanhaecke

**Affiliations:** 1 Faculty of Bioscience Engineering, Laboratory of Biochemistry and Brewing, Ghent University, Valentin Vaerwyckweg 1, B-9000 Ghent, Belgium; Anita.Vanlandschoot@ugent.be (A.V.L.); Hellen.Watson@ugent.be (H.W.); 2 Faculty of Veterinary Medicine, Department of Veterinary Public Health and Food Safety, Laboratory of Chemical Analysis, Ghent University, 133 Salisburylaan, B-9820 Merelbeke, Belgium; Lynn.Vanhaecke@ugent.be; 3 Faculty of Science and Technology, Department of Biosciences and Food Sciences, University College Ghent, Valentin Vaerwyckweg 1, B-9000 Ghent, Belgium; Dana.Vanderputten@hogent.be

**Keywords:** (conjugated) plant sterols, beverages, cholesterol-lowering, ergosterol, anti-aging, coronary heart disease, health claims, anti-inflammatory, anti-carcinogenic

## Abstract

To address the ever-growing group of health-conscious consumers, more and more nutritional and health claims are being used on food products. Nevertheless, only very few food constituents, including plant sterols, have been appointed an approved health claim (European Commission and Food and Drugs Administration). Plant sterols are part of those limited lists of approved compounds for their cholesterol-lowering properties but have been praised for their anti-inflammatory and anti-carcinogenic properties as well. Despite this indisputable reputation, direct quantitative data is still lacking for naturally present (conjugated) plant sterols in beverages. This study aimed to fill this gap by applying a validated extraction and UPLC-MS/MS detection method to a diverse range of everyday plant-based beverages. β-sitosterol-β-d-glucoside (BSSG) showed to be by far the most abundant sterol in all beverages studied, with concentrations up to 60–90 mg per 100 mL in plant-based milk alternatives and fresh fruit juices. Ergosterol (provitamin D_2_) could be found in beers (0.8–6.1 µg per 100 mL, from the yeast) and occasionally in juices (17–29 µg per 100 mL). Overall, the results demonstrated that the concentrations of water-soluble sterol conjugates have been underestimated significantly and that specific plant-based beverages can be good, low-fat sources of these plant sterols.

## 1. Introduction

Over the last decade, the beverage industry has taken a serious jump into the future by introducing a broad range of new, convenient, natural, and functional beverages. These beverages, often called health drinks, include (iced) teas and juices but also shakes and “super drinks” such as pomegranate juice or *Aloe vera* extract–based drinks. For most of these products, a range of different nutrition and health claims are being formulated on the label and in advertisements. These claims stimulate the consumer to purchase these particular beverages in order to increase their personal health status. Popular health claims are “free from” claims such as gluten-free or lactose-free but also vegan, organic, “helps to prevent coronary heart disease” and “lowers cholesterol” claims are being used quite often. To streamline these claims, FDA (Food and Drug Administration) and EC (European Commission) rules have been adopted on the use of nutrition and health claims on foods. Up until now, only very few compounds have been appointed an approved health claim by both organizations. Plant sterols are part of that limited list of approved compounds for their cholesterol-lowering properties (FDA Health Claim; Phytosterols and Risk of Coronary Heart Disease) (EFSA, Article 14 (1)(a) “Reduction of disease risk” of the Regulation on nutrition and health claims 1924/2006). Less well-known, but perhaps even more promising, are a range of other suggested health benefits related to the consumption of plant sterols such as anti-carcinogenic, anti-inflammatory, and anti-oxidative effects [1,2,3,4,5,6]. Despite this general interest, quantitative data on the concentration of these sterols in day-to-day consumption goods and especially beverages are extremely scarce.

**Figure 1:**
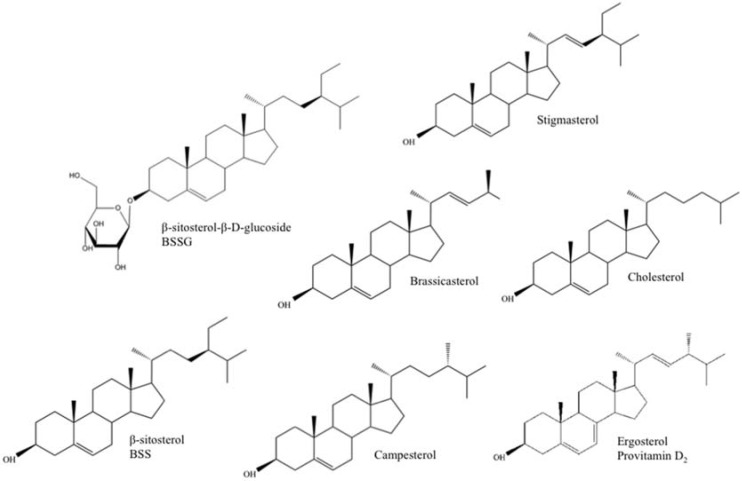
Chemical structure of the main free and conjugated plant sterols (campesterol, stigmasterol, brassicasterol, β-sitosterol (BSS), and β-sitosterol-β-d-glucoside (BSSG)), cholesterol (animal sterol), and ergosterol (fungal/yeast sterol, provitamine D_2_).

Plant sterols, or phytosterols, are one of the main constituents of plant membranes, playing an important role in cell membrane stability and as signal transducers [7]. Ergosterol (provitamin D_2_), on the other hand, takes up a similar role in protozoa and fungi (e.g., yeast) and is a provitamin form of vitamin D_2_ or ergocalciferol. Exposure of ergosterol to ultraviolet (UV) light causes a photochemical reaction that produces vitamin D_2_. This happens naturally to a certain extent, and quite often, mushrooms are irradiated after harvest to increase their Vitamin D_2_ content [8]. Fungi are also grown industrially so that ergosterol can be extracted and converted to Vitamin D_2_ for sale as a dietary supplement and food additive [9]. Chemically, ergosterol (provitamin D_2_) and plant sterols are very alike and similar to their human and animal counterpart, cholesterol (Figure 1). They contain a stereo-specific oriented methyl or ethyl substitution at the C24 position of the sterol side chain and, in the case of stigmasterol, ergosterol, and brassicasterol, an additional double bound between C22 and C23 [4,10] (Table 1). Upon human consumption, these structural and functional resemblances allow plant sterols and ergosterol to interfere with cholesterol absorption in the intestinal tract through displacement of cholesterol from the micelles and/or competition with cholesterol binding proteins. As a result, low-density lipoprotein (LDL) cholesterol levels will decrease, lowering the risk for coronary heart failure [11,12].

The European Food Safety Authority (EFSA) and FDA concluded that, relative to a placebo, blood LDL cholesterol levels can be reduced by 7 to 12.5% if a person consumes 1.5 to 3 grams of plant sterols and stanols (expressed as free sterols) a day (EFSA claim, article 14(1)(a)) [13,14]. A recent meta-analysis by Ras et al. (2014) (124 studies, 202 stratas) extended these findings; they found that plant sterol intakes of 0.6 to 3.3 g per day gradually reduce LDL-cholesterol concentrations by, on average, 6 to 12% [15]. The cholesterol-lowering effect is usually established within two to three weeks after diet change and could be sustained for months [16]. No significant alterations in high-density-lipoprotein (HDL)-cholesterol (the “good” cholesterol), or triglycerides in general, were reported. Effectiveness of this approach has been positively tested in hypercholesteraemic patients as well as in individuals with normal cholesterol levels [17]. 

FDA and EC rules on novel food and novel food ingredients (in force since 1997) require all new ingredients to go through an applicant-specific authorization procedure that involves a rigorous safety assessment before they can be placed on the market (Regulation (EC) No 258/97, concerning novel foods and novel food ingredients). Under these rules, approval has been given for the addition of plant sterols in a range of foods, but these are mostly high-fat products such as yellow fat spreads, dairy products (e.g., yogurt), mayonnaise, and salad dressings. Unsaturated forms of plant sterols, phytostanols, have also been added to food. An example hereof is a non-fatty alternative, chewable plant stanol ester gum, for which efficacy has been confirmed recently [18]. Nevertheless, high concentrations of phytostanols are not natural, as high concentrations of phytostanols are very rare in most plants (with the exemption of a few cereal species and their derived products such as rice oil) [7]. 

Natural water-soluble (glycosidic) plant sterols could be a good alternative for these (fatty) phytostanol- and phytosterol-enriched products [19], especially if they can be obtained from easy to consume low-fat and low-energy natural food sources such as beverages. Unfortunately, only very limited direct, quantitative data is available on the natural presence of (glycosidic) plant sterols. Racette et al. (2009) already noted that glycosylated plant sterols are often excluded from sterol analysis, mostly due to the lack of standards and analytical difficulties [20]. In their study, total plant sterol content, including glycosides, was computed indirectly as the sum of the individual plant sterols determined by double (acidic and alkalic) hydrolysis. Their indirect analyses showed that glycosylated plant sterols (in general) comprised 20% of total plant sterols, in different diets. They also suggested that nuts, seeds, legumes, wheat germ, whole grains, bran, fruit, and vegetables could be important sources of glycosylated plant sterols. The presence of BSSG in dietary supplements and (fatty) foods such as nuts and wheat has also been touched upon by Phillips et al. (2005). The highest concentrations found back then, using indirect detection, were in flaxseed and soybean (up to 11 mg per 100 g dry weight, DW) [21]. Muller et al. (2007) suggested that BSS(G) and ergosterol from beer can compete with cholesterol during protein binding and as such prevent cholesterol uptake. However, he did not succeed in quantifying the true amounts of BSS(G) present in beers [22]. 

Therefore, this manuscript aimed to extend an extraction method and UPLC-MS/MS detection method, which was recently optimized and fully validated according to EC 2002/657 guidelines and Association of Analytical Chemists (AOAC) MS criteria, with these two compounds of interest (β-sitosterol-β-d-glucoside and ergosterol) (Multiple Reaction Monitoring, MRM) [23]. Campesterol, stigmasterol, brassicasterol, ergosterol (provitamine D_2_), BSSG, and BSS concentrations were determined in a broad range of plant-based beverages, including a variety of (concentrate-based) juices, vegetable juices, beers, teas, malt-based (non-alcoholic) drinks, and plant-based milk alternatives (e.g., oat or soy beverages). Particular attention was payed to sample selection to cover a range of drinks that is as broad as possible. Plant extract–containing sodas were also included for comparison. Overall nutritional values and other (non-)beneficial compounds used in the formulation were also summarized (e.g., concentrations of proteins, vitamins, and minerals present).

## 2. Materials and Methods

Chloroform (analytical grade) and HPLC grade methanol (Methanol Optima^®^) were purchased from Fisher Scientific (Leicestershire, UK). Methanol (analytical grade) was purchased from VWR (Merck, Darmstadt, Germany). HPLC grade, ultrapure (UP) water was acquired from an in-house water purification system (Arium^®^ 611UV, Sartorius Stedium Biotech, VWR, Haasrode, Belgium). Cholesterol (≥99%, from lanolin), β-sitosterol (BSS) (≥97%, from soy beans), brassicasterol (≥95%, from semisynthetic), provitamin D_2_ (ergosterol) (≥97%, European Pharmacopoeia Reference Standard), and stigmasterol (≥97%, Supelco, Certified Reference Material) were purchased from Sigma Aldrich (St-Louis, Missouri, USA). Campesterol (≥98%, from seeds of *Brassica campestris*) was obtained from Wuhan ChemFaces Biochemical Co., Ltd. (Wuhan, Hubei, China). β-sitosterol-β-d-glucoside (BSSG) (≥95%, from semisynthetic) was purchased from Neuroquest (Halifax, NS, Canada). Stock solutions of each component (500 or 200 ng/µL) and dilutions up to 1 ng/µL were made in HPLC grade methanol. All solutions were kept at 4 °C and protected from direct light (brown flasks and additional aluminum foil coat).

Beverages were purchased from different suppliers/producers including Oat-ly AB (Mälmo, Sweden), 2Food (Soesterberg, The Netherlands), Paulaner Brauerei GmbH & Co. KG (Münich, Germany), AB Inbev (Leuven, Belgium), Olgerdin Egill Skallagrímsson Brewery (Reykjavik, Iceland), Ghent University College (Ghent, Belgium), The Coca-Cola Company (Ghent, Belgium), Melitta België n.v. (Lokeren, Belgium), Dream^TM^ Hain Celestial Group, Inc. (Aalter, Belgium), Continental Foods Belgium (Puurs, Belgium), Pepsico Belux BVBA/SPRL (Zaventem, Belgium). Delhaize Le Lion/De Leeuw (Brussel, Belgium), Alpro, The WhiteWave Foods Company (Wevelgem, Belgium), Forever Living Products (Scottsdale, AZ, USA), Tao family (Ternat, Belgium), NV Brasseries Alken-Maes SA (Malines/Opwijk, Belgium), Haacht Brewery plc (Boortmoorbeek, Belgium), Carlsberg Breweries A/S (Copenhagen, Denmark), Palm breweries (Steenhuffel, Belgium), Brasserie du Bocq (Purnode, Belgium), Duvel Moortgat NV (Puurs, Belgium), Omer Vander Ghinste Brewery (Bellegem, Belgium), Nestle SA (Vevey, Switzerland), and Unilever (Brussel, Belgium).

Statistical model designs were used to optimize the general analytical extraction procedure. Dependent variables that might significantly affect the extraction efficiency were screened with a fractional factorial D-optimal design. These variables were selected on the basis of a literature survey and further optimization of only the influential variables was performed through response surface modeling (RSM) (Modde Pro 12, Umetrics software, Sartorium Stedim Biotech, Umeå, Sweden). The optimal sample volume for liquid samples (beverages) was determined using an additional small-scale full factorial design, and 5 mL was found to be the optimal sample volume, both in relative response per mL and S/N [23] (Table 2).

An ultra-high performance liquid chromatography tandem mass spectrometry (UPLC-MS/MS) detection method was used for the quantification of free plant sterols, ergosterol, and BSSG in a single run. Previously, this method was fully validated for quantification of campesterol, BSS, stigmasterol, ergosterol, and brassicasterol [23]. Preliminary experiments showed that this method is also suitable for quantification of BSSG. Separation was carried out using an Accela^TM^ High Speed LC (Thermo Fisher Scientific, San Jose, CA, USA) equipped with a Thermo Fisher Scientific™ Hypersil GOLD™ C18 Column (particle size: 1.9 μm, 50 × 2.1 mm I.D.). The mobile phases used were ultra-pure water (solvent A) and methanol (LC-MS grade, solvent B). All analytes could be accurately separated in a total run time of less than 10 min (Table 3). The gradient started with a linear gradient of 90% solvent B (methanol) for the first 2 min, increasing to 100% at 5.5 min, and then held at 100% for 1.5 min (up to 7 min). Afterward, the column was allowed to equilibrate at the initial conditions of 10% A and 90% B for 2 min. The divert valve was used to load the detector from 1.0 to 4.5 min. Scheduling was used to increase sensitivity, by limiting the detection window for each analyte to 0.6 min before and after the expected retention time. Detection was carried out on a TSQ Vantage triple stage quadrupole mass spectrometer equipped with an atmospheric pressure chemical ionization probe (APCI) (Thermo Fisher Scientific, San Jose, CA, USA). Injection volumes were 10 µL each and the APCI source was operated in the positive ion mode. The discharge current was set at ±4 µA. The sheath, sweep and auxiliary gas pressures were set at 20, 2, and 10 arbitrary units, respectively, the capillary temperature at 300 °C, and the vaporizer temperature at 320 °C. The collision gas pressure was kept at 1.5 mTorr, and the cycle time was 0.8 s. Data were acquired in the selected/multiple reaction-monitoring (SRM/MRM) mode. All specified product ions (Table 3) were used for peak integration, ion ratio determinations, and quantification purposes.

Area ratios were calculated relative to the internal standard (ISTD) cholesterol, which was added to both calibration and unknown samples, to compensate for losses during sample preparation and/or variation of the analytical analysis. Cholesterol was considered a suitable internal standard as no significant endogenous concentrations are present in the samples of interest (plant-based) and cholesterol is very similar to the calibrated analytes (Figure 1; Table 1 and Table 3), chemically and in retention time but nevertheless chromatographically distinguishable and less expensive than isotopically labeled standards. Applying this method to other samples that, contrary to the samples analyzed in the current study, do contain significant concentrations of endogenous cholesterol would imply the use of another internal standard (e.g., 5α-cholestan-7β-ol or a deuterated (glycosidic) plant sterol).

Of each beverage, at least three non-fortified samples were extracted together with a nine-point matrix-matched calibration curve (≥12 samples per matrix), constructed based upon nine fortification levels (0, 0.25, 0.5, 1, 2, 4, 6, 8, and 10 times the minimal expected endogenous concentration of each plant sterol individually). The minimal expected endogenous concentration was preliminary determined based upon calculated expected endogenous concentrations and standard addition (analysis of matrix matched samples with known added concentrations of plant sterols). Calculations combined available reference values for solid ingredients with their expected minimal contribution (%) to the different beverages. All samples were run twice, to take into account analytical variance, and mean (*n* = 6) ± standard deviation of these duplicate runs are reported in Table 4 and Table 5.

For almost half of the samples (*n* = 19/49), endogenous concentrations of BSSG and/or BSS turned out to be too high to be able to include fortified samples with two to ten times the endogenous concentrations. For those matrices, additional calibration curves were made in diluted samples and additional diluted non-fortified samples were analyzed (diluted 4- to 30-fold with UP water, depending on the matrix). For all samples, endogenous plant sterol concentrations were determined by fitting the compounds’ area ratio of non-fortified samples into the corresponding calibration curve. For diluted samples, the concentrations in undiluted samples were recalculated afterward.

## 3. Results

### 3.1. Data Analysis and Quality Assurance of the Analytical Method: Limits of Detection and Quantification

Lower limits of quantification (LLOQs) in solvent (90:10 methanol:H_2_O) for the method used were between 0.5 and 1.5 ng per mL. In (diluted) beverages, the LLOQ was 0.5–3.0 µg per 100 mL for liquid samples, depending on the general composition of the sample and the compound of interest. In general, fatty and protein rich beverages (e.g., soy and oat beverage) hampered the detection of very low concentrations of plant sterols. Fortunately, most of these beverages were also relatively high in plant sterols. Previous results showed that the limits of detection (LODs) for solid matrices for the different compounds were between 10 and 30 µg per 100 g and LLOQs were between 20 and 100 µg per 100 g [23].

### 3.2. Quantification of Cholesterol-Lowering (Conjugated) Plant Sterols and Ergosterol (Provitamin D_2_) with UPLC-MS/MS

All UPLC-MS/MS determined concentrations of BSS, BSSG, stigmasterol campesterol, brassicasterol, and ergosterol (provitamin D_2_) in a diverse range of beverages (fruit juices, vegetable juices, plant-based milk alternatives, gel, soft drinks, teas, (non-alcoholic) malt-based drinks, and beers) have been summarized in Table 4 and Table 5. 

## 4. Discussion

Where possible, obtained plant sterol concentrations were compared to previously obtained thin layer chromatography (TLC), gas- or liquid chromatography—mass spectrometry (LC/GC-MS) and GC-FID (Flame Ionization Detector) results available in literature.

### 4.1. Fruit Juices

BSSG was present in very high concentrations (up to >90 mg per 100 mL). These concentrations are much higher than the mean concentrations determined in corresponding plants in the past, through indirect analysis. These results showed that in general only 20% of the plant sterols found in edible plants (<10 mg per 100 mL) were conjugated [20]. It is thus very likely that glucose-conjugated plant sterols are currently being underestimated in solid matrices, where they are tightly matrix-bound. However, due to their water-soluble nature, they are enriched throughout the production process of plant-based beverages. In addition, it can’t be excluded that the previously used indirect method based upon chemical hydrolysis [20] was not sufficient to release conjugated plant sterols from the matrix and complete hydrolysis of the β-glycosidic bound at the same time. 

BSS was the most abundant free plant sterol found, in line with mean free plant concentrations in higher plants, where BSS accounted for 50 to 80% of the total amount of plant sterols [24]. The highest concentration of BSS (5.3 ± 2.2 mg per 100 mL) was measured in the fresh orange-banana juice. As a comparison, this is 2.1 and 2.5 times higher than the concentration of BSS in the pomegranate juice and the multifruit-carrot juice, respectively. The lowest concentration of BSS was found in the concentrated orange and apple juice (13 and 25 time lower, respectively).

Interestingly, the two fruit juices that contained orange juice as a main ingredient (80% in fresh orange-banana juice, 100% in the concentrate-based orange juice; Supplementary Materials Table S1) contained very similar ratios of BSSG:BSS:campesterol, but the fresh orange-banana juice had 12 to 14 times higher concentrations. The concentration difference for stigmasterol was 26-fold, but that can be a direct consequence of the 20% banana content. Bananas are typically very high in stigmasterol; containing up to 200 mg per 100 g dry weight of banana pseudostem (pulp) [25,26,27]. 

The large difference between both orange juice based beverages can be explained by looking into detail at the results obtained for the apple and orange concentrate based juices, which happened to be produced by the same producer. Their plant sterol concentrations were found to be in linear correlation with the reported concentrations in the corresponding fruits (orange 23–24 mg and apple 12 mg per 100 g fresh weight with 11 mg BSS, 0.3 mg campesterol and 0.05 mg stigmasterol) [23,28,29,30,31]. For both juices (100% fruit), only around 2% of the initial plant sterol content of the fruits seemed to end up in the concentrate-based juices (g to mL), which suggests they used similar production processes for both concentrate-based juices, who were unfortunately equally incapable of containing plant sterols into the final juices. This illustrates the possible loss of beneficial compounds during the concentration process. Previous research has shown that the industrial production process can have a significant influence on the final concentrations (e.g., vitamin C and phenols). Previous research also showed that commercial squeezing of oranges extracted 22% more phenols than hand squeezing, however, the freezing process caused a dramatic decrease in phenols and the concentration process caused a mild precipitation of these compounds to the juice cloud [32]. Similar results have been reported for phenols in apple juices, but this could be prevented by using initial high temperature-short time (HTST) treatment and diffusion extraction instead of pressing [33]. Additional research will be needed to elucidate the exact influence of different fruit juice concentrate production processes on the concentrations of plant sterols in the final juice.

In pomegranate juice another peculiar result was detected in the chromatogram for stigmasterol. Stigmasterol could not be quantified as an intense interfering peak appeared at a retention time very close to the retention time of stigmasterol (with the same precursor and fragment ion masses). Upon addition of stigmasterol (matrix-matched calibration curve), it was confirmed that this peak was not stigmasterol. It seems that pomegranate (juice) contains a specific compound/plant sterol, very similar to stigmasterol, or a specific conjugated form of stigmasterol that deconjugates upon ionization (see also 4.6 Other water-soluble glucose-conjugated plant sterols). This illustrates the difficulties caused by the lack of analytical standards for (conjugated) plant sterols. Over 200 different types of plant sterols have been described [7], but for only a very limited number of those plant sterols analytical grade (≥95% purity) standards are available. As such, it is very likely that analytical results still underestimate the contribution of, mostly exotic, novel fruits and other parts of plants that were previously not used for consumption (novel foods list EU 1997, e.g., pomegranate, chia seeds, Aloe vera). Especially as a revision of the novel foods directive is due to come into force in January 2018; the approval process will be significantly shortened and simplified (and thus less expensive) for new fruits or juices as long as a 25 years history of use can be shown in the country of origin (new Directive for Traditional Foods, EC Regulation No. 2015/2283). The introduction of these new fruits or juices onto the market will hamper the accurate detection of plant sterols even more, as these fruits will be accompanied by less well known plant sterols, wherefore no analytical standards are available. Additional research will also be needed to evaluate if these less well-known plant sterols exhibit the same health benefits.

The tropical multifruit-carrot juice was also concentrate-based (18% orange, 19% pine apple, 5% carrot and 3% other fruits including passion fruit, Supplementary Materials Table S1). Orange, pine apple and carrots are moderately high in plant sterols, containing respectively 23, 17 and 12 mg of plant sterols per 100 g edible fresh weight, while passion fruit is high in plant sterols (44 mg per 100 g) [28,31]. However, as with most exotic beverages, the true amount of passion fruit blended into the drink was too low to contribute its beneficial effects (<1%). Nevertheless, the concentrations of plant sterols in this juice were very high, similar to the concentrations measured in fresh juices. When having a look at the results for the carrot juice (4.2 Vegetable juices) it can be noted that the concentrations measured are very alike. So it seems that despite its low contribution percentage-wise, carrot juice does make a strong contribution to the final plant sterol concentrations in the juice. Upon extraction it was already visible that this juice was visually more turbid and more viscous (thicker) than the 100% orange concentrate-based juice. This observation was supported by the ingredient list. This beverage included both juice and sauce concentrates (45%), and not just juice concentrate. Including fruit sauces implies that the cell material rich solid fraction (pulp) is included into the beverage.

No brassicasterol was found in either of these juices, but that is in line with what could be expected, as their ingredient lists (Supplementary Materials Table S1) did not include plants from the Brassicaceae family or products derived from these plants.

### 4.2. Vegetable Juices

In line with the concentrations measured in fruit juices, BSSG was detected in the highest concentrations, but the difference with BSS was less profound. Respectively, 1.0 ± 0.1 and approximately 10 mg per 100 mL BSS and BSSG were found in the vegetable drinks analyzed. A possible explanation for this is that fruits are typically a lot higher in carbohydrates, and monosaccharides in particular, than vegetables (e.g., orange and pine apple 9 g, apple 10 g, banana 15 g per 100 g fresh weight (FW) versus tomatoes and lettuce 2 g, carrot 6 g, broccoli and cucumber 1 g per 100 g FW), hence the formation of high concentrations of glucose conjugates seems more likely (FDA nutrition facts raw fruits and raw vegetables poster, 2016). This sugar content difference is still detectable in the corresponding juices with mean sugar concentrations of 10 ± 3 and 5 ± 2 g sugar per 100 mL fruit juices and vegetable juices, respectively (Supplementary Materials Table S1), and thus reflected in the conjugated plant sterol concentrations measured. Mixed vegetable juice (b) is a good example thereof, as it contains almost twice as much sugars (5.7 g per 100 mL) as the other mixed vegetable juice (a) (2.7 g per 100 mL), and as a result contained a 2-fold greater amount of BSSG (12 ± 3 versus 6.2 ± 0.5 mg per 100 mL, respectively). The main difference in the ingredient list of both juices is that juice (b) contains less tomato and celeriac/onion juice (2 g sugar per 100 g FW) and more carrot juice (6 g sugar per 100 g FW).

Interestingly, and as touched upon in 4.1 Fruit juices, carrot juice showed to be particularly high in plant sterols, containing two to three times more plant sterols than the other vegetable juices. However, carrots are not particularly high in plant sterols, suggesting that the juice production technology and the true amount of carrots used per mL play an important role as well. Generally, fresh carrots are peeled with steam or mechanical peelers, chopped and eventually cooked to have a better extraction of the juice. The cooked carrots are mashed in a turbo extractor and then cooled and stored in treatment tanks where enzymes can be added (increasing juice yield yet reducing carotene content) [34]. After the enzyme treatment the obtained carrot juice is pasteurized for enzyme inactivation. After that, the carrot juice passes through the decanter to remove fibers and can be concentrated for transport. The main difference with the production of other juices is that these are typically not cooked before juice extraction, which might influence the plant sterol concentrations in the resulting juice. This effect is reflected in the concentrations of plant sterols found in beetroot juice and carrot juice. One of the main differences between both juices is that fresh beets are used to produce beetroot juice, while carrots are boiled before juice extraction. The plant sterol content of their respective raw materials, beetroot and carrot, are very similar (17.1 and 15.3 mg per 100 g FW) [28], yet carrot juice contained at least six times more plant sterols. Despite this lack of plant sterols, beetroot (juice) is being put forward as a promising therapeutic treatment in a range of clinical pathologies associated with oxidative stress and inflammation, due to the presence of other anti-oxidative constituents [35].

Botanically, tomatoes would be categorized as fruits, but as this study has been performed from a consumer’s point of view, they have been added to the vegetable juices group. The plant sterol concentrations measured were also more in line with the vegetable juices group than with the, generally higher in plant sterols, fruit juices group. Both mixed vegetable juices (a) and (b) also contained high concentrations of tomato juice (respectively 86% and 79.6% versus 99% in the tomato juice), which was reflected by the plant sterol concentrations measured in each of them. Surprisingly, vegetable juice (b) contained ergosterol (29 ± 4 µg per 100 mL), just like the pomegranate juice (17 ± 4 µg per 100 mL) (fruit juices group). The most plausible explanation for this is that the vegetables/fruits used for the production of these juices were contaminated with fungi. Parsi and Gorecki (2006) described how ergosterol could be used as an indicator for fungal biomass [36]. Upon visual fungal outgrowth they detected respectively 140 mg and 17 mg ergosterol per 100 g on moldy bread and maple leaves infected with powdery mildew, putting into perspective the concentrations measured here. Multiple authors have reported additional expectable concentrations of ergosterol per 100 g cells (DW) (Table 6) but cell mass estimations based upon concentrations of ergosterol detected will always be rough estimates, as the concentration of ergosterol present in fungal/yeast cells is dependent of light, age of the cell and growth conditions [37]. In any case, these very low concentrations of ergosterol measured did not contribute significantly to the overall intake of sterols.

### 4.3. Plant-Based Milk Alternatives

High concentrations of BSS (up to 4 mg per 100 mL) and especially BSSG (up to 78 mg per 100 mL) were detected in the almond, oats and cashew base milk alternatives. The rice and rice-coconut based beverages, on the other hand, only contained low concentrations of plant sterols (<5 mg per mL in total). The soy based beverage contained higher concentrations of campesterol (1.3 ± 0.3 mg per 100 mL) and stigmasterol (0.9 ± 0.1 mg per 100 mL), and moderate concentrations of BSS and BSSG, respectively 2.5 ± 0.5 and 4.9 ± 1.9 mg per 100 mL. The high concentrations of campesterol and stigmasterol in soy are however in line with results obtained in a study by Yamaya et al. (2007) [40]. This study showed that the content of plant sterols ranged from 202 and 843 µg per g soy bean. The highest amounts were found in soybeans with high lipid content. BSS, campesterol, and stigmasterol were the main plant sterols found at the proportions of 43–67%, 17–34%, and 10–30%, respectively. The concentrations measured in the soy beverage are in line with these ranges, but at the higher end of the range for campesterol (respectively 52%, 27% and 21% in the soy based beverage). Another difference that immediately pops out of the list of results is the significant difference between the two almond based beverages. Almond beverage (a) contained particularly higher concentrations of BSSG (78 ± 14 mg per 100 mL) than almond beverage (b) (13 ± 2 mg per 100 mL), and lower concentrations of stigmasterol (<0.03 mg per 100 mL) versus (1.9 ± 0.1 mg per 100 mL). An important difference between these beverages however is that almond beverage (a) was based upon unroasted almonds, while for almond beverage (b) roasted almonds were used and beverage (a) was sweetened, while beverage (b) was not.

Brassicasterol is typically found in plants belonging to the Brassicaceae family. The family contains different edible plants such as *Brassica oleracea* (e.g., broccoli, cabbage and cauliflower), *Brassica nigra* and *Sinapis alba* (black and white mustard seeds). Also part of this family are the canola oilseeds producing members of the species *Brassica rapa*, including *Brassica rapa* subspecies oleifera (field mustard) and *Brassica napus* (rapeseed), and the mustard subspecies of *Brassica juncea* (e.g., green and brown mustard). Canola oil producers claim that the total amount of free plant sterols in edible (low erucic acid) canola oils ranges from 0.63% to 0.71% with 10.8–16.2% brassicasterol, which would translate to 76–112 mg per 100 g for different cultivars (Canola Council of Canada, canola oil chemical properties, 2017). Mo et al. (2013) confirmed that canola oil can contain very high levels of brassicasterol, and other plant sterols, compared to other edible oils but they detected a more realistic concentration of 48.8 mg brassicasterol per 100 g [41]. Piironen et al. (2000) reported similar concentrations between 55 and 73 mg per 100 g [42]. Compared to that, concentrations of brassicasterol found in different vegetables such as cabbage, Brussels sprouts and broccoli are very low, ranging between 0.2 and 2.0 mg per 100 g [30]. Oat beverage (b), listing canola oil on its ingredient list (percentage used not listed), showed to contain 217 ± 12 µg brassicasterol per 100 mL. This concentration translates to 5 (or more) g canola oil per L oat beverage. The total fat percentage of the oat beverage is 1.5% (originating from the oats, 7–8% fat; and canola oil, 100% fat), proving that brassicasterol is a good indicator for the amount of canola oil used. The addition of canola oil is also reflected in the concentrations of other plant sterols found in oat beverage (b). Especially the high concentration of campesterol found in this beverage (1.10 ± 0.06 versus 0.48 ± 0.05 mg per 100 mL in oat beverage (a)) could be attributed to canola oil. 

Interestingly, two other plant-based milk alternatives (soy and rice based) also contained traces of brassicasterol (respectively 10 ± 4 and 4.6 ± 0.4 µg per 100 mL), although they did not list canola oil as an ingredient. Given the low concentration of brassicasterol in the rice beverage it is most likely that sunflower oil, that is part of the rice beverage’s ingredient list, (unintentionally) got mixed with canola/mustard seed oil (<1:50 ratio). The soy beverage result is even more intriguing, as no oil at all was mentioned in the ingredient list. The integrity of this beverage might be questionable. This suspicion is strengthened by the high concentration of campesterol and stigmasterol found in this soy beverage, suggesting that a (campesterol rich) oil might have been added after all. Sterol profiling has already been used to unravel adulteration of other (expensive) oils such as extra virgin olive oil with other cheaper oils [43,44,45], but these results show that with this sensitive method it is even possible to trace back the botanical origin of oils in processed end products such as beverages. 

### 4.4. Gel, Sodas, Teas and Non-Alcoholic Malt-Based Drinks

Most of the teas, sodas, and (non-alcoholic) malt-based drinks contained only low concentrations of plant sterols (10 to >100-fold lower than the juices and plant-based milk alternatives discussed earlier) (Table 4 and Table 5, orange color code). However, some exceptions should be noted. The *Aloe vera* gel–based drink showed to be moderately high in BSSG (17 ± 5 mg per 100 mL), yet low in the other plant sterols. The orange juice concentrate-based lemonade contained similar concentrations of plant sterols as the concentrate-based orange juice analyzed, illustrating the dilution effect and/or loss of plant sterols throughout both production processes. The other two malt-based (biolemonades) analyzed were slightly higher in plant sterols compared to other sodas (and teas/non-alcoholic beers/malt drinks) but still far less plant sterol rich than fruit and vegetable juices and plant-based milk alternatives.

### 4.5. Beers

The only available data on plant sterols in beer are results from Muller et al. (2007) and Rapota and Tyrsin (2015) [22,46]. Both indicated that BSS from malt and hop could compete with cholesterol for protein binding and uptake. Rapota and Tyrsin (2015) were able to prove the qualitative presence of plant sterols in malt and hop, but no quantitative data were reported [46]. Our own preliminary data showed that brewer’s hop and malting barley contain, respectively, >140 and >50 mg free plant sterols per 100 g DW [23]. Muller et al. (2007) analyzed a few beer samples (*n* = 4), proving the presence of BSS in beer, but the results were not quantitative [22]. Throughout the current study, BSSG, BSS, campesterol, ergosterol, and stigmasterol could be detected and quantified in a variety of different beers (Table 5). In general, plant sterol concentrations in beer were very low (on average 10 times lower than concentrations in juices and milk alternatives). Nevertheless, these results did allow us to unravel correlations between the production process of the different beer types (technologies used) and the concentrations measured in the end beer.

The wheat beers (*n* = 4) contained the highest concentration of plant sterols, probably due to the lack of end filtration in this type of beer, which allows grain residues to remain in the final beer. This is also reflected by the turbidity of these beers compared to lagers and ales. The H90 EBC (European Brewery Convention units) turbidity, an indicator for the presence of sub 1 µm particles such as proteins ranged between 96 and >100 EBC in wheat beers, versus 12 ± 5 EBC and 0.55 ± 0.17 for ales (*n* = 4) and lagers (*n* = 4), respectively (*p* < 0.001). H25 EBC, indicative for larger particles such as yeast and diatomaceous earth, was 72 ± 14 EBC in wheat beers, versus 21 ± 12 EBC and 0.25 ± 0.05 EBC for lagers and ales, respectively (*p* < 0.001). One of the ales (ale (a)) was not filtered either, so this beer was excluded from the pool of ales. Its turbidity was indeed closer to the turbidity of the wheat beers (H90 47 ± 2 EBC and H25 71 ± 3 EBC). The opposite was true for ale (e), which includes very extensive removal of the cold break and end filtration in its production process. This is reflected by both the low turbidity (H90 9 ± 1 EBC, H25 13 ± 1 EBC) and low concentrations of plant sterols measured, three times lower than the other ales (Table 5). In general, however, top fermented ales contained higher concentrations of plant sterols compared to lager beers. This can be related to the higher original extract (16.4 ± 1.2 P), which is directly linked to the grain bill (amount of grain used), unless sugar or other carbon sources are being added (Supplementary Materials Table S1). The mean original extract was significantly lower in lagers (11.3 ± 0.4 P, *p* < 0.001) and wheat beers (10.3 ± 0.9 P, *p* < 0.001). 

Interestingly, plant sterol concentrations measured in alcoholic beers and their non-alcoholic counterparts were significantly lower in the latter, suggesting that different production processes and less grain were used to produce the non-alcoholic alternatives, further reducing their plant sterol content. This is also reflected by the significantly lower mean original extract values measured in non-alcoholic beers compared to their alcoholic counterparts (6.9 ± 0.4 P versus 10.8 ± 0.6, *p* < 0.001). Ergosterol was only detected in the wheat beers and ales (with bottle refermentation), not in the non-alcoholic or lager beers. However, the concentrations were very low, showing that only very limited amounts of the yeast and its ergosterol end up in the glass. For ales with refermentation in the bottle, the yeast adheres to the bottle; therefore, ergosterol is not consumed. 

### 4.6. Other Water-Soluble Glucose-Conjugated Plant Sterols

**Figure 2:**
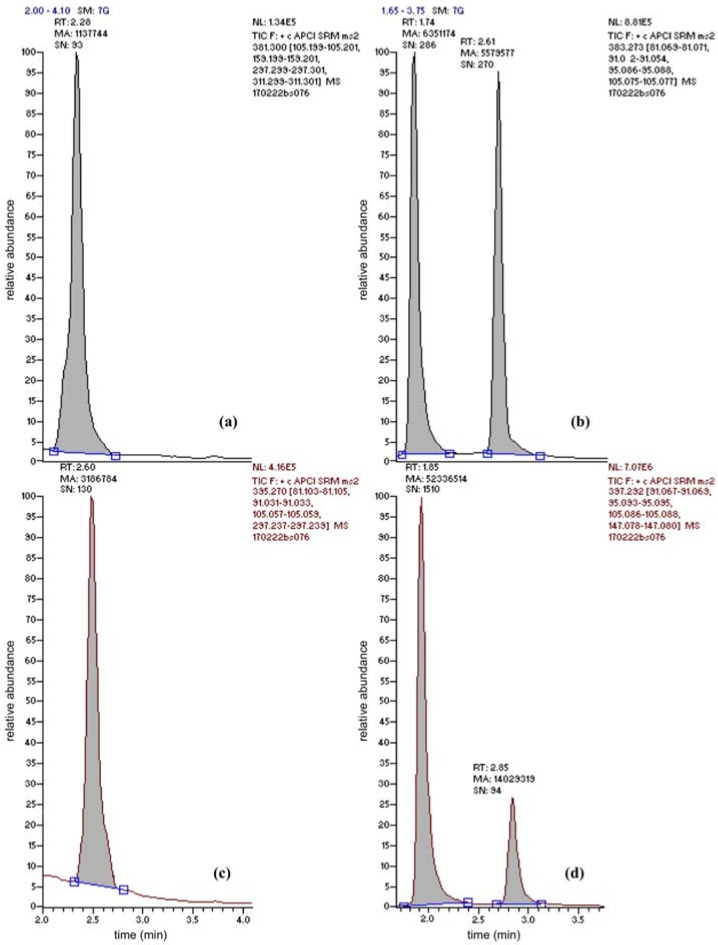
Chromatogram obtained using UPLC–MS/MS after the injection (10 µL) of a four times diluted organic oat beverage extract. This beverage contained 217 ± 12 µg brassicasterol (**a**), 1.1 ± 0.1 mg campesterol (**b**), <15 µg stigmasterol per 100 mL (**c**) and 3.9 ± 0.7 mg BSS and 33 ± 4 mg BSSG (**d**). Panel (**b**) also clearly illustrates the presence of high concentrations of campesterol-β-d-glucoside (retention time 1.74 min, *m*/*z* 383.3).

BSSG was the only conjugated plant sterol for which an analytical standard (≥95% NMR purity) could be acquired. Nevertheless, this standard allowed understanding the fate of conjugated plant sterols in general. Due to the presence of the polar glucose conjugate the retention on the apolar column was less than for the free sterols (retention times for BSS and BSSG were 2.90 min and 1.80 min, respectively). A mean absolute retention time difference of 1.1 ± 0.1 min between both peaks could be determined. Glucose-conjugated plant sterols will thus arrive into the ionization source significantly earlier than their free counterparts. Upon ionization (APCI), the β-glycosidic bound is broken down; the main precursor ion measured in the first quadrupole (Q1) matches the precursor ion for free BSS. Upon Q2 fragmentation, the same product ions and product ion ratios could be found in Q3. Interestingly, when broadening the detection window, free campesterol was found to be proceeded by a second peak as well, at a very similar relative retention time difference (0.43 ± 0.01 for BSS and BSSG and 0.38 ± 0.01 for campesterol and “campesterol-glucoside”). Taking into account the mass spectral and product ion ratio match of this peak with campesterol precursor and product ions, it could be concluded that this peak is most likely campesterol-β-d-glucoside. In line with the results obtained for BSSG, the peak area of campesterol-β-d-glucoside is at least as high as the peak for free campesterol (Figure 2, top right campesterol-glucoside and campesterol, bottom right BSSG and BSS). This campesterol-β-d-glucoside peak was found in all beverages analyzed. It can be concluded that current methodologies to measure total plant sterol content underestimate the contribution of these glycosidic conjugates to the total plant sterol content.

## 5. Conclusions

This study aimed to quantify free and conjugated plant sterols and ergosterol in a broad range of plant-based (health) drinks. Concentrations of water-soluble glycosidic phytosterols (e.g., BSSG) showed to be much higher than what could have been expected from concentrations previously (indirectly) determined in solid foods such as grains, fruits, and vegetables. Plant-based milk alternatives and fresh juices for example showed to contain up to 90 mg BSSG per 100 mL. Due to their water-soluble nature, these sterols may have been enriched throughout the liquid extraction process used to produce these beverages. Most concentrate based beverages and extracts on the other hand only contained low concentrations of plant sterols. In addition, previously used extraction and chemical hydrolysis protocols might not have sufficed to release all conjugated plant sterols from the matrix and complete hydrolysis of the β-glycosidic bound at the same time. In light of the ever-growing market of health-conscious consumers, one should be looking into more detail at production processes to increase enrichment of (conjugated) plant sterols were possible. Another possibility is to further expand the possibilities of the addition of water-soluble glycosylated plant sterols to low energy foods such as drinks instead of esterified plant sterols to fatty food matrices.

## Figures and Tables

**Table 1:** Chemical similarities and structural differences between cholesterol, ergosterol (provitamin D_2_) and plant sterols. Listed characteristics include mean mass, molecular formula, number of double bounds, position of double bounds, and alkyl group present at C24 (none, methyl, or ethyl).

Sterol Name	Mean Mass (Da)	Molecular Formula	Structural Characteristics		
			Double Bounds	Position Double Bounds	Alkyl Group at C24
**Cholesterol**	386.654	C_27_H_46_O	1	C5–C6	/
**Ergosterol** **(Provitamin D_2_)**	396.648	C_28_H_44_O	3	C5–C6 C7–C8 C22–C23	Methyl
**β-sitosterol-β-d-glucoside (BSSG)**	576.847	C_35_H_60_O_6_	1	C5–C6	Ethyl
**β-sitosterol** **(BSS)**	414.707	C_29_H_50_O	1	C5–C6	Ethyl
**Brassicasterol**	398.675	C_28_H_46_O	2	C5–C6 C22–C23	Methyl
**Stigmasterol**	412.691	C_29_H_48_O	2	C5–C6 C22–C23	Ethyl
**Campesterol**	400.691	C_28_H_48_O	1	C5–C6	Methyl

**Table 2:** Summary of the validated extraction protocol to extract plant sterols and ergosterol from a diverse range of beverages [23].

**1.**	5 mL (diluted) ^∆^ beverage in a 50 mL tube
**2.**	Addition of cholesterol (100 µL, 10 ng or 50 ng per µL)
**3.**	(Calibration samples: fortified with different plant sterols)
**4.**	Liquid-liquid extraction with 8 mL chloroform:methanol (2:1)
**5.**	Vortex (30 s) + ultrasonication * (10 min)
**6.**	Centrifugation (4400× *g*, 10 min)
**7.**	Cottonwool filter
**8.**	Second liquid-liquid extraction (repeat step 2 to 5)
**9.**	1 mL fresh chloroform:methanol added to the filter (filter wash out)
**10.**	Transfer 2 mL extract to new tube
**11.**	15–20 min drying (under liquid N_2_, 46 °C)
**12.**	180 μL methanol (vortex 30 s, ultrasonication * 10 min)
**13.**	20 μL ultrapure H_2_O (vortex 30 s, ultrasonication * 3 min, vortex 30 s)
**14.**	Centrifugation (12,300× *g*, 10 min)
**15.**	Transfer 150 µL to plastic LC-MS vial with insert for analysis

^∆^ Samples were diluted if the first results showed that endogenous concentrations were too high to be able to include calibration points containing two to ten times the endogenous concentration (mostly for BSSG and BSS); * Ultrasonication: power 100, frequency 80 kHz.

**Table 3:** Selected/multiple reaction monitoring (SRM/MRM) specifics for all compounds of interest: precursor ions, product ions (as *m*/*z*, mass over charge), absolute and relative retention time (RT, in minutes, min), appropriate S-Lens amplitude (volt, V), and the corresponding collision energy (CE, in electron volt, eV).

Analyte	Precursor Ion	Product Ions	Mean Relative Ion Abundancy *	Retention Time (Relative)	S-Lens Voltage	Collision Energy
	(*m*/*z*)	(*m*/*z*)	(%)	(min)	(V)	(eV)
**β-Sitosterol-β-d-glucoside BSSG**	397.3	91	70	1.80 (0.77)	88	47
		95	73			35
		105	100			40
		147	93			28
**Ergosterol ** **Provitamine D_2_**	379.3	69	78	2.04 (0.87)	120	23
		91	100			53
		105	90			34
		15	82			24
**Brassicasterol**	381.3	105	100	2.27 (0.97)	82	43
		159	67			23
		297	93			14
		311	40			13
**Cholesterol** **internal standard**	369.3	91	83	2.35 (1.00)	84	52
		95	69			34
		105	100			40
**Campesterol**	383.3	81	67	2.61 (1.11)	86	35
		91	85			49
		95	74			34
		105	100			43
**Stigmasterol**	395.3	81	64	2.63 (1.12)	59	37
		91	91			52
		105	100			44
		297	90			18
**β-sitosterol** **BSS**	397.3	91	70	2.90 (1.23)	88	47
		95	73			35
		105	100			40
		147	93			28

* In solvent, at full width half maximum (FWHM) and relative to the product ion with the highest intensity.

**Table 4:** UPLC-MS/MS determined concentrations of BSS, BSSG, stigmasterol, campesterol, brassicasterol, and ergosterol (provitamin D_2_) in a diverse range of beverages (fruit juices, vegetable juices, plant-based milk alternatives, gel, sodas, teas, and (non-alcoholic) malt-based drinks and beers).

Category	Product Name	mg per 100 mL		µg per 100 mL				
		BSS	BSSG	Brassicasterol	Campesterol	Stigmasterol	Ergosterol	
**Fruit juices**	Apple juice	0.21 ± 0.01	4.1 ± 1.2 *	NF (<0.75)	27 ± 3	2.6 ± 0.4	ND	
	Orange juice	0.42 ± 0.09	8.3 ± 2.3 *	NF (<1.5)	71 ± 12	23 ± 3	ND	
	Pomegranate juice	2.1 ± 0.3	32 ± 7	NF (<3)	139 ± 18	<30	17 ± 6	
	Multifruit-carrot juice	2.5 ± 0.2	16 ± 3	NF (<3)	607 ± 12	224 ± 10	ND	
	Fresh orange-banana juice	5.3 ± 2.2	>90	NF (<3)	846 ± 93	610 ± 35	NF (<3)	
**Vegetable juices**	Tomato juice	0.36 ± 0.02	4.4 ± 0.5	NF (<2)	155 ± 10	331 ± 27	ND	
	Mixed vegetable juice (a)	0.74 ± 0.05	6.2 ± 0.5	NF (<3)	242 ± 24	596 ± 64	ND	
	Mixed vegetable juice (b)	0.72 ± 0.10	12 ± 3	NF (<3.75)	177 ± 25	359 ± 72	29 ± 4	
	Beetroot juice	0.42 ± 0.07	7.3 ± 1.2	NF (<3)	47 ± 8	40 ± 3	NF (<3)	
	Carrot juice	2.7 ± 0.4	18 ± 4	NF (<3)	677 ± 68	1270 ± 65	NF (<3)	
**Plant-based milk alternatives**	Coconut-rice	0.51 ± 0.07	2.8 ± 0.9	NF (<3)	72 ± 10	76 ± 13	ND	
	Rice	1.4 ± 0.1	2.4 ± 0.6	10 ± 3	260 ± 28	234 ± 23	ND	
	Soy	2.5 ± 0.5	4.9 ± 2.1	4.6 ± 0.4	1290 ± 291	998 ± 111	ND	
	Cashew	2.7 ± 0.4	>60	NF (<3)	279 ± 44	15 ± 1	NF (<3)	
	Almond (a) unroasted	2.6 ± 0.6	78 ± 14	NF (<3)	101 ± 30	<30	ND	
	Almond (b) roasted	2.5 ± 0.1	13 ± 2	NF (<2)	62 ± 4	1915 ± 109	ND	
	Oat (a)	2.1 ± 0.2	26 ± 4	NF (<3)	475 ± 30	182 ± 16	ND	
	Oat (b)	3.9 ± 0.7	33 ± 4	217 ± 12	1098 ± 61	<15	NF (<3)	
**Gel**	*Aloe vera* gel beverage	0.22 ± 0.03	17 ± 5	NF (<0.75)	23 ± 5	2.2 ± 0.9	ND	
**Sodas**	Lemonade (a) (orange)	0.48 ± 0.04	1.5 ± 0.3*	NF (<0.75)	73 ± 3	19 ± 2	NF (<0.75)	
	Biolemonade (a) (elderberry)	0.19 ± 0.02	2.3 ± 0.7	NF (<1.5)	24 ± 2	52 ± 15	NF (<0.75)	
	Biolemonade (b) (ginger-orange)	0.17 ± 0.05	2.5 ± 0.7	NF (<0.75)	24 ± 3	4.3 ± 1.0	NF (<0.75)	
	Soda with plant extract (a)	0.05 ± 0.01	1.1 ± 0.2	NF (<0.75)	8.0 ± 1.7	1.3 ± 0.3	NF (<0.75)	
	Soda with plant extract (b) (stevia)	0.05 ± 0.01	0.60 ± 0.10	NF (<0.75)	7.3 ± 1.1	1.9 ± 0.3	NF (<0.75)	
	Soda with plant extract (c) (peach)	0.06 ± 0.01	1.5 ± 0.5	NF (<0.75)	5.8 ± 1.2	0.80 ± 0.31	NF (<0.75)	
**Teas**	Tea infusion (a)	0.08 ± 0.01	0.67 ± 0.12	NF (<0.75)	9.2 ± 1.7	1.1 ± 0.3	NF (<0.75)	
	Tea infusion (b)	0.06 ± 0.01	0.65 ± 0.17	NF (<0.75)	9.6 ± 1.3	1.5 ± 0.2	NF (<0.75)	
	Tea infusion (c)	0.06 ± 0.01	0.68 ± 0.12	NF (<0.75)	6.9 ± 0.8	2.3 ± 0.2	NF (<0.75)	
	Iced tea (b)	0.06 ± 0.01	0.93 ± 0.32	NF (<0.75)	8.3 ± 1.6	5.8 ± 0.8	NF (<0.75)	
	Iced tea (c)	0.05 ± 0.01	0.84 ± 0.08	NF (<0.75)	6.7 ± 0.8	9.5 ± 1.4	NF (<0.75)	

* Using a group specific calibration curve from respectively pomegranate juice (fruit juices) or lager (a) (beers); 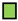 Indicates a plant-based beverage that can be considered a good source of free and conjugated plant sterols; 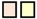 Indicates a plant-based beverage that contains only moderate (yellow) or low concentrations (orange) of plant sterols. NF, not found; ND, not determined.

**Table 5:** UPLC-MS/MS determined concentrations of BSS, BSSG, stigmasterol, campesterol, brassicasterol, and ergosterol (provitamin D_2_) in a diverse range of beverages (fruit juices, vegetable juices, plant-based milk alternatives, gel, sodas, teas, and (non-alcoholic) malt-based drinks and beers).

Category	Product Name	mg per 100 mL		µg per 100 mL				
		BSS	BSSG	Brassicasterol	Campesterol	Stigmasterol	Ergosterol	
**Non- alcoholic malt drinks**	Chinese malt drink	0.07 ± 0.02	0.95 ± 0.33	NF (<0.75)	6.6 ± 2.1	<LOQ (<2)	ND	
	Icelandic malt drink	0.14 ± 0.04	2.74 ± 1.31	NF (<1.5)	19 ± 4	2.4 ± 0.9	ND	
	Non-alcoholic lager (a)	0.04 ± 0.01	0.50 ± 0.07	NF (<0.75)	6.8 ± 2.4	1.0 ± 0.4	NF (<0.75)	
	Non-alcoholic lager (b)	0.07 ± 0.03	1.6 ± 0.5	NF (<0.75)	11 ± 3	1.9 ± 0.8	ND	
	Non-alcoholic wheat beer (a)	0.07 ± 0.02	1.0 ± 0.1	NF (<0.75)	7.7 ± 1.9	0.88 ± 0.35	ND	
	Non-alcoholic wheat beer (b)	0.12 ± 0.03	1.4 ± 0.2 *	NF (<0.75)	14 ± 3	2.1 ± 0.4	ND	
**Beers**	Lager (a)	0.20 ± 0.04	2.2 ± 0.3	NF (<0.75)	24 ± 5	3 ± 1	NF (<0.75)	
	Lager (b)	0.26 ± 0.02	1.7 ± 0.3 *	NF (<0.75)	31 ± 4	5 ± 1	ND	
	Lager (c)	0.25 ± 0.04	1.2 ± 0.3 *	NF (<0.75)	39 ± 5	7 ± 1	ND	
	Lager (d)	0.23 ± 0.03	1.7 ± 0.3 *	NF (<0.75)	23 ± 4	5 ± 1	ND	
	Wheat beer (a)	0.28 ± 0.04	2.8 ± 0.4 *	NF (<0.75)	52 ± 3	6 ± 1	4.1 ± 0.4	
	Wheat beer (b)	0.38 ± 0.09	3.2 ± 0.6 *	NF (<0.75)	53 ± 11	7 ± 2	ND	
	Wheat beer (c)	0.26 ± 0.02	1.9 ± 0.3 *	NF (<0.75)	30 ± 2	3 ± 1	ND	
	Wheat beer (d)	0.27 ± 0.04	3.4 ± 0.4 *	NF (<0.75)	37 ± 6	5 ± 1	ND	
	Ale (bottle fermented) (a)	0.37 ± 0.05	2.0 ± 0.3 *	NF (<0.75)	49 ± 7	9 ± 1	0.80 ± 0.18	
	Ale (bottle fermented) (b)	0.23 ± 0.03	4.5 ± 0.5 *	NF (<0.75)	36 ± 4	6 ± 1	6.0 ± 0.3	
	Ale (bottle fermented) (c)	0.25 ± 0.03	2.5 ± 0.5 *	NF (<0.75)	36 ± 4	4 ± 1	ND	
	Ale (bottle fermented) (d)	0.23 ± 0.03	1.3 ± 0.1 *	NF (<0.75)	7 ± 2	2 ± 1	4.6 ± 0.8	
	Ale (bottle fermented) (e)	0.09 ± 0.02	0.9 ± 0.1 *	NF (<0.75)	36 ± 4	6 ± 1	ND	

* Using a group specific calibration curve from respectively pomegranate juice (fruit juices) or lager (a) (beers); 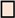 Indicates a plant-based beverage that contains only moderate (yellow) or low concentrations (orange) of plant sterols. NF, not found; ND, not determined.

**Table 6:** Concentrations of provitamine D_2_ (ergosterol) measured in yeast and fungi (mg per 100 g dry weight, DW). Ranked according to concentration [36,37,38,39].

Species	Ergosterol (mg per 100 g DW)
Yeast	
*Cryptococcus albidus*	4200 ± 1200
*Rhodotorulamucilaginosa*	3700 ± 760
*Rhodotorulaminuta*	3700 ± 630
*Saccharomycescerevisiae*	400–2000
Fungi	
*Acremoniumfurcatum*	1400 ± 780
*Stachybotryschartarum*	1200 ± 520
*Aspergillus versicolor*	1100 ± 1500
*Penicilliumbrevicompactum*	580 ± 1300
*Cladosporiumcladosporioides*	560 ± 1100
*Aureobasidiumpullulans*	260 ± 1600

## Supplementary Materials

The following are available online at www.mdpi.com/2072-6643/10/1/21/s1, Supplementary Materials Table S1: Detailed composition of the diverse beverages analyzed, including beverage category (group), beverage name, health claims listed, main ingredients, ingredients percentage, added sugars and/or sweeteners, vitamins, minerals, stabilizers and thickeners used, energy, fat, carbohydrates, fibers, proteins, salt content, and alcohol percentage. Upon request, the authors can also provide information on the producer and batch number of a beverage sample of interest.
