# Supplementary material for: Plant-Based Beverages as Good Sources of Free and Glycosidic Plant Sterols
Source: Nutrients. 2017 Dec 29;10(1):21. doi: 10.3390/nu10010021 (PMC5793249; doi:10.3390/nu10010021)
Supplement: Supplementary file 1 [file nutrients-10-00021-s001.pdf]

Table S1. *Supplementary material*. Detailed composition of the diverse beverages analyzed, including beverage category (group), beverage name, health claims listed, main ingredients, ingredients percentage, added sugars and/or sweeteners, vitamins, minerals, stabilizers and thickeners used, energy, fat, carbohydrates, fibers, proteins, salt content and alcohol percentage.

Category	Name	Health claims	Main ingredients (not mentioned seperately -->)	%	Added sugar sweeteners	Vitamins*	µg per 100 mL	Minerals	mg per 100 mL	Stabilzers Thickeners	Energy (kcal per 100 mL)	Fats (g per 100 mL) (of which saturated)	Carbohydrates (g per 100 mL) (of which sugars)	Fibres (g per 100 mL)	Proteins (g per 100 mL)	Salt (g per 100 mL)	Alcohol (V/V%)
Fruit juices	Apple juice	*100% fruit*	Water<br>Apple juice (concentrated)	100	/	Vit C	20000	/		/	47	0.0 (0.0)	11.3 (11.3)	/	0.0	0	0.0
	Orange juice	*100% orange juice*<br>*Unsweetened*	Water<br>Orange juice (concentrated)	100	/	Vit C	20000	/		/	47	0.0 (0.0)	11.3 (11.3)	/	0.7	0	0.0
	Pomegranate juice	Gluten-free<br>Dairy-free<br>Soy-free<br>Egg-free**<br>*100% fruit*	Pomegranate juice	100	/	Vit C	?	/		/	51	0.0 (0.0)	12.3 (12.3)	0.0	0.3	0.0	0.0
	Tropical multifruit-carrot juice	Low energy (low-calorie)<br>Source of vit A<br>Source of vit C<br>Source of vit E	Water<br>Orange juice (and sauce) (concentrated)<br>Pine apple juice (and sauce) (concentrated)<br>Kiwi juice (and sauce) (concentrated)<br>Banana juice (and sauce) (concentrated)<br>Passion fruit juice (and sauce) (concentrated)<br>Mango juice (and sauce) (concentrated)<br>Lime juice (and sauce) (concentrated)<br>Papaya juice (and sauce) (concentrated)<br>Carrot juice (concentrated)<br>Aromas<br>Citric acid (acidic regulator)	19<br>18<br>3<br>5	Acesulfame K<br>Sodium saccharin<br>Natrium cyclamate	Vit C<br>Vit E<br>Provitamine A	40000<br>6000<br>400	/		/	18	0.0 (0.0)	4.0 (4.0)	0	0.3	0	0.0
	Fresh orange-banana juice	Source of fibre (3 g/100 g or 1.5 g/ 100 kcal)<br>*100% fruit*<br>*Freshly squeezed*	Orange juice<br>Banana	80<br>20	/	/		/		/	50	0.0 (0.0)	11.2 (11.2)	0.8	0.8	0	0.0
Vegetable juices	Tomato juice	/	Tomato juice (concentrated)<br>Lemon juice (concentrated)<br>Salt	>99	/	/		/		/	14	<0.2 (<0.1)	2.6 (2.6)	0.0	0.8	0.61	0.0
	Mixed vegetable juice (a)	Source of fibre (3 g/100 g or 1.5 g/ 100 kcal)<br>Gluten-free<br>Lactose-free<br>No added sugars<br>No preservatives<br>No colorants<br>*100% vegetal*	Tomato juice (concentrated)<br>Carrot juice (concentrated)<br>Celery (concentrated)<br>Beetroot juice (concentrated)<br>Parsley (concentrated)<br>Lettuce juice (concentrated)<br>Watercress juice (concentrated)<br>Spinach (concentrated)<br>Salt<br>Spice mix (contains celery)	86<br>13	/	/		/		/	18	0.1 (0.0)	3.0 (2.7)	0.9	0.9	0.68	0.0
	Mixed vegetable juice (b)	100% vegetal	Tomato juice (concentrated)<br>Celeriac juice<br>Carrot juice<br>Lemon juice (concentrated)<br>Onion juice<br>Beetroot<br>Salt<br>Chervil<br>Basil<br>Spice mix<br>Beet red (coloring agent)<br>Pepper	79.8<br>19.2<br>0.2	/	/		/		/	25	0.0 (0.0)	5.9 (5.9)	0.3	0.5	0.75	0.0
	Beetroot juice	Organic (BE-BIO-O2 and O1 EU/non-EU)	Beetroot juice<br>Acerola juice	99<br>1	/	/		/		/	37	0.1 (0.0)	7.9 (7.9)	0.2	1.1	0.08	0.0
	Carrot juice	Organic (BE-BIO-O2 and O1 EU/non-EU)	Carrot juice (concentrated)<br>Lemon juice (concentrated)	99<br>1	/	/		/		/	22	0.0 (0.0)	4.7 (4.7)	0.3	0.6	0.17	0.0

* Some beverages do contain ascorbic acid as an antioxidant, but if the exact dose per 100 mL is not mentioned on the label it is not included here, only in the main ingredients list

** Does not contain: tree nuts, soybeans, peanuts, eggs, sulfite, mollusks, crustaceans, celery, lupines, milk, fish, mustard, sesame, gluten

Table S1. *Supplementary material*. Detailed composition of the diverse beverages analyzed, including beverage category (group), beverage name, health claims listed, main ingredients, ingredients percentage, added sugars and/or sweeteners, vitamins, minerals, stabilizers and thickeners used, energy, fat, carbohydrates, fibers, proteins, salt content and alcohol percentage.

Category	Name	Health claims	Main ingredients (not mentioned seperately -->)	%	Added sugar sweeteners	Vitamins*	µg per 100 mL	Minerals	mg per 100 mL	Stabilzers Thickeners	Energy (kcal per 100 mL)	Fats (g per 100 mL) (of which saturated)	Carbohydrates (g per 100 mL) (of which sugars)	Fibres (g per 100 mL)	Proteins (g per 100 mL)	Salt (g per 100 mL)	Alcohol (V/V%)
Plant-based milk alternatives	Coconut/rice	Low energy (low-calorie)<br>Gluten-free<br>Dairy-free<br>Milk-free<br>Lactose-free<br>No added sugars<br>100% vegetal<br>Source of vit B12<br>Naturally low in saturated fat<br>*Easy to digest*	Water<br>Coconut milk (coconut cream and water)<br>Rice<br>Tricalcium phosphate<br>Salt (sea)<br>Aromas	5.3<br>3.3	/	Vit B2<br>Vit D2	0,38<br>0,75	Ca	120	Carrageenan<br>Guar gum<br>Xanthan gum	20	0.9 (0.9)	2.7 (1.9)	0.0	0.1	0.13	0.0
	Rice	Vegetarian<br>Source of calcium<br>Source of vit B2<br>Source of vit B12<br>Source of vit D2<br>*Extra <3*	Water<br>Rice<br>Sunflower oil<br>Calcium carbonate<br>Salt	13.4	/	Vit B2<br>Vit B12<br>Vit D	210<br>0,38<br>0,75	Ca	120	Gellan gum	58	1.0 (0.1)	12.0 (2.9)	0.3	0.2	0.1	0.0
	Soy	Dairy-free<br>Milk-free<br>Gluten-free<br>Lactose-free<br>100% vegetal<br>Naturally low in saturated fat<br>Low in fat<br>No colorants<br>Free of preservatives<br>Source of protein<br>Source of vit B12<br>Source of vit D<br>Source of vit B2<br>Source of calcium<br>Vegan<br>*Easy to digest*	Water<br>Soy beans (pealed)<br>Tricalcium phosphate<br>Monopotassium phosphate (acid regulator)<br>Salt<br>Aromas	5.9	Sugar	B2<br>Vit B12<br>Vit D2	210<br>0,38<br>0,75	Ca	120	Gellan gum	39	1.8 (0.3)	2.5 (2.5)	0.5	3.0	0.11	0.0
	Cashew	Dairy-free<br>Gluten-free<br>Low in fat<br>Low in sugar<br>Source of vit D<br>Source of vit B2<br>Source of vit B12<br>Source of calcium	Water<br>Cashew nuts<br>Tricalcium phosphate<br>Salt<br>Sunflower lecithine (emulsifier)	3.1	Sugar	Vit B2<br>Vit B12<br>Vit D2<br>Vit E	210<br>0,38<br>0,75<br>1800	Ca	120	Locust bean gum<br>Gellan gum	23	1.1 (0.2)	2.6 (2.0)	0.2	0.5	0.13	0.0
	Almond (a)	Dairy-free<br>Gluten-free<br>Low in fat<br>Source of vit E<br>Source of vit D	Water<br>Almond<br>Tricalcium phosphate<br>Salt<br>Sunflower lecithine (emulsifier)	2.1	Sugar	Vit B2<br>Vit B12<br>Vit E<br>Vit D2	210<br>0.38<br>1800<br>0.75	Ca	120	Locust bean gum<br>Gellan gum	24	1.1 (0.1)	3.0 (3.0)	0.2	0.5	0.14	0.0
	Almond (b)	Dairy-free<br>Gluten-free<br>No added sugars<br>Source of vit E<br>Source of vit D<br>Source of vit B2<br>Source of vit B12<br>Source of calcium	Water<br>Almond<br>Tricalcium phosphate<br>Salt<br>Sunflower lecithine (emulsifier)<br>Aromas	2.1	/	Vit B2<br>Vit B12<br>Vit D2<br>Vit E	210<br>0.38<br>0.75<br>1800	Ca	120	Locust bean gum<br>Gellan gum	13	1.3 (0.1)	0.1 (0.1)	0.2	0.5	0.14	0.0
	Oat (a)	*Natural ingredients*<br>No added sugars<br>Soy-free<br>Source of fibre (3 g/100 g or 1.5 g/ 100 kcal)<br>Milk-free<br>Lactose-free<br>Source of calcium<br>Source of vit B12<br>Source of vit D<br>Vegan<br>Vegetarian<br>Low in fat<br>Halal	Water<br>Oats<br>Sunflower oil<br>Tricalcium phosphate<br>Salt (sea)	16	/	vit D2<br>Vit B12	0.75<br>0.375	Ca	120	Acacia gum (gum arabic)	52	1.4 (0.3)	8.9 (5.9)	1.0	0.4	0.12	0.0
	Oat (b)	Milk-free<br>Soy-free<br>100% vegetal<br>Source of fibre (3 g/100 g or 1.5 g/ 100 kcal)<br>0.4 g beta-glucan/100 mL<br>*Doesn't contain coconut, rice or nuts*	Water<br>Oats<br>Canola oil<br>Calcium carbonate<br>Tricalcium phosphate (and other)<br>Salt	10	/	Vit D2<br>Riboflavine<br>Vit B12	1.5<br>210<br>0.38	Ca	120	/	45	1.5 (0.2)	6.5 (4.0)	0.8	1.0	0.1	0.0
Gel	Aloë Vera	Gluten-free<br>Vegan<br>Vegetarian<br>Halal<br>*Beneficial for maintaining a healthy digestive system*	Stabilized Aloe Vera inner leaf gel<br>Citric acid (acidic regulator)<br>Ascorbic acid (antioxidant)<br>Potassium benzoate (preservative)<br>Tocopherol (antioxidant)	96	Sorbitol	/		/		Xanthan gum	17	0.0 (0.0)	5.0 (0.0)	/	0.0	0.25	0.0

* Some beverages do contain ascorbic acid as an antioxidant, but if the exact dose per 100 mL is not mentioned on the label it is not included here, only in the main ingredients list

Table S1. *Supplementary material*. Detailed composition of the diverse beverages analyzed, including beverage category (group), beverage name, health claims listed, main ingredients, ingredients percentage, added sugars and/or sweeteners, vitamins, minerals, stabilizers and thickeners used, energy, fat, carbohydrates, fibers, proteins, salt content and alcohol percentage.

Category	Name	Health claims	Main ingredients (not mentioned seperately -->)	%	Added sugar sweeteners	Vitamins*	µg per 100 mL	Minerals	mg per 100 mL	Stabilzers Thickeners	Energy (kcal per 100 mL)	Fats (g per 100 mL) (of which saturated)	Carbohydrates (g per 100 mL) (of which sugars)	Fibres (g per 100 mL)	Proteins (g per 100 mL)	Salt (g per 100 mL)	Alcohol (V/V%)
Sodas	Lemonade (orange)	*With orange-juice*	Orange juice (concentrated)<br>Citric acid (acidic regulator)<br>Sodium citrate<br>Potassium sorbate (preservative)<br>Aromas<br>Ascorbic acid (antioxidant)<br>Carrot juice (concentrated)	6	Sugar	/		/		Guar gum	48	0.0 (0.0)	11.7 (11.7)	/	0.0	0	0.0
	Biolemonade (a) (elderberry)	Gluten-free<br>Biological origin<br>Kosher<br>Vegetarian<br>Vegan<br>No preservatives<br>No colorants<br>*Non-alcoholic*	Water<br>Barley malt<br>Elderberry juice (concentrated)<br>Carbondioxide<br>Aromas<br>Calcium carbonate<br>Magnesium carbonate	 <br>2	Sugar	/		/		/	22	<0.5 (<0.1)	5.0 (5.0)	/	<0.5	<0.1	0.0
	Biolemonade (b) (orange-ginger)	Organic (DE-OKO-003 EU/non-EU)<br>Kosher<br>Vegetarian<br>Vegan<br>No preservatives<br>No colorants<br>Gluten-free<br>*Non-alcoholic*	Water<br>Barley malt<br>Elderberry juice (concentrated)<br>Carbondioxide<br>Aromas<br>Ginger extract<br>Orange juice (extract)<br>Calcium carbonate<br>Magnesium carbonate	 <br>2<br><br><br><br>2	Sugar	/		/		/	19	<0.5 (<0.1)	4.3 (4.3)	/	<0.5	<0.01	0.0
	Soda with plant extract (a)	/	Water (carbonated)<br>Caramel (E150d)<br>Phosphoric acid<br>Aromas (plant extracts)<br>Caffeine	 <br><br><br>0.010	Sugar	/		/		*not labeled*	42	0.0 (0.0)	10.6 (10.6)	/	0.0	0.013	0.0
	Soda with plant extract (b) (stevia sweetened)	*45% less sugar*<br>*45% less calories*	Water (carbonated)<br>Caramel (E150d)<br>Phosphoric acid<br>Aromas (plant extracts)<br>Caffeine	 <br><br><br>0.008	Sugar<br>Steviolglycosides	/		/		*not labeled*	27	0.0 (0.0)	5.8 (5.8)	/	0.0	0	0.0
	Soda with plant extract (c) (peach)	/	Water<br>Citric acid (acidic regulator)<br>Carrot juice (concentrated)<br>Zwarte bessenconcentraat<br>Aromas<br>Salt<br>Sodium citrate<br>Potassium phosphate<br>Calcium phosphate<br>Carotenes (coloring agent)		Sugar	Vit B6	110	/		Acacia gum (gum arabic)<br>Wood rosins	31	0.0 (0.0)	7.4 (7.4)	/	0.0	0.06	0.0
Teas	Tea infusion-based drink (a)	Low energy (low-calorie)<br>Natural ingredients	Fruit tea extract (water and herbal mix)<br>Cranberry juice<br>Apple berry juice (concentrated)<br>Apple juice (concentrated)<br>Elderberry juice (concentrated)<br>citron juice (concentrated)<br>acerola juice (concentrated)<br>Carbondioxide<br>Kombucha starter culture<br>*Lactobacillus*	 5.1	Sugar (beetroot)	/		/		/	19	<0.1 (<0.1)	4.6 (4.5)	/	0.1	0.008	0.0
	Tea infusion-based drink (b)	Low energy (low-calorie)<br>No added sugars<br>No preservatives<br>No colorants<br>No flavor-enhancers<br>Natural ingredients	Water<br>fruit extracts<br>Lemon juice (concentrated)<br>Elder blossom (extract)<br>Tea-extract (rooibos)<br>Prume juice (concentrate)<br>Ascorbic acid (antioxidant)<br>passion flower extract	 <br>2<br>0.04<br>0.28<br>0.2<br><br>0.003	*Not labeled*	Vit C	1600	/		/	19	<0.5 (<0.1)	5.0 (5.0)	<0.5	<0.5	<0.01	0.0
	Tea infusion-based drink (c)	No artificial flavours<br>No colorants<br>No preservatives<br><br>*made with real brewed tea*	Water<br>Tea-extract (black tea)<br>Citric acid (acidic regulator)<br>Ascorbic acid (antioxidant)<br>Aromas	95<br>0.1	Sugar<br>Steviolglycosides	/		/		/	19	<0.5 (<0.1)	4.6 (4.5)	0.0	<0.5	<0.01	0.0
	Iced tea (a)	No colorants<br>Low energy (low-calorie)	Water (carbonated)<br>Tea-extract<br>citric acid and malic acid (acidulant)<br>tripotassium citrate (acidic regulator)<br>Lemon juice (concentrated)<br>Aroma<br>Ascorbic acid (antioxidant)	 0.3<br><br><br>0.1	Sugar (invert sugar)<br>Fructose<br>Steviolglycosides	/		/		/	20	<0.5 (< 0.1)	4.6 (4.5)	/	<0.5	0.11	0.0
	Iced tea (b)	/	Water<br>Citric acid (acidic regulator)<br>Sodium citrate (acidic regulator)<br>Tea-extracts<br>Potassium sorbate (preservative)<br>Aromas<br>Ascorbic acid (antioxidant)		Steviolglycosides<br>Sugar<br>Fructose	/		/		/	21	0.0 (0.0)	4.8 (4.8)	/	0.0	0.05	0.0

* Some beverages do contain ascorbic acid as an antioxidant, but if the exact dose per 100 mL is not mentioned on the label it is not included here, only in the main ingredients list

Table S1. *Supplementary material*. Detailed composition of the diverse beverages analyzed, including beverage category (group), beverage name, health claims listed, main ingredients, ingredients percentage, added sugars and/or sweeteners, vitamins, minerals, stabilizers and thickeners used, energy, fat, carbohydrates, fibers, proteins, salt content and alcohol percentage.

Category	Name	Health claims	Main ingredients (not mentioned seperately -->)	%	Added sugar sweeteners	Vitamins*	µg per 100 mL	Minerals	mg per 100 mL	Stabilzers Thickeners	Energy (kcal per 100 mL)	Fats (g per 100 mL) (of which saturated)	Carbohydrates (g per 100 mL) (of which sugars)	Fibres (g per 100 mL)	Proteins (g per 100 mL)	Salt (g per 100 mL)	Alcohol (V/V%)
**Non-alcoholic malt drinks**	Chinese grain drink	100% vegetal	Water<br>Grain extract<br>Malt extract (concentrate)<br>Antioxidant (E300)<br>Acidulant (E500)	45<br>0.15	/	/		/		/	0	0.0 (0.0)	0.0 (0.0)	/	0.0	0.017	0.0
	Icelandic malt drink	*Non-alcoholic*	Water<br>Barley<br>Hop<br>Yeast<br>Caramel (E150c)<br>Licorice		Sugar	/		/		/	59	0.0 (0.0)	14.0 (8-11.4)	/	0.5-1	0.01	0.0
	Non-alcoholic lager (a)	*Non-alcoholic*	Water<br>Barley malt<br>Corn<br>Hop<br>Caramel (E150c)		/	/		/		/	26	0.0 (0.0)	6.1 (3.9)	/	0.3	< 0.1	<0.25
	Non-alcoholic lager (b)	*Non-alcoholic*	Water<br>Barley malt<br>Wheat malt<br>Hop<br>Hop extract		/	/		/		/	26	0.0 (0.0)	6.1 (6.1)	/	0.3	<0.1	0.4
	Non-alcoholic wheat beer (a)	*Non-alcoholic*	Water<br>Barley malt<br>Wheat<br>Aromas<br>Carbondioxide<br>Citric acid (acidic regulator)<br>Coriander<br>Dried orange peals<br>Hop<br>Apple extract		Sugar	/		/		/	27	0.0 (0.0)	7.4 (3.7)	/	0.5	<0.1	0.0
	Non-alcoholic wheat beer (b)	Contains vit B12<br>Low energy (low-calorie)<br>*Contains folacin*<br>*Natural*<br>*Isotonic*<br>*Non-alcoholic*	Water<br>Barley malt<br>Wheat malt<br>Hop<br>Yeast<br>Carbondioxide		/	Folic acid/ vit B9<br>Vit B12	29.6<br>0.14	/		/	20	0.0 (0.0)	4.5 (1.3)	/	0.6	<0.1	<0.5
**Beers**	Lager (a)	/	Water<br>Barley malt<br>Corn<br>Hop<br>Caramel (E150c)		/	/		/		/	43	0.0 (0.0)	3.2 (0.1)	/	0.4	<0.1	5.2
	Lager (b)	/	Water<br>Barley malt<br>Hop		/	/		/		/	/	/	/	/	/	<0.1	5.2
	Lager (c)	*Reinheitsgebot*	Water<br>Barley malt<br>Hop extract		/	/		/		/	32	0 (0)	2.0 (0.0)	/	0.3	<0.1	5.0
	Lager (d)	/	Water<br>Barley malt<br>Hop		Sugar	/		/		/	/	/	/	/	/	<0.1	4.9
	Wheat beer (a)	/	Water<br>Barley malt<br>Wheat	 60 (rel.)<br>40 (rel.)	/	/		/		/	/	/	/	/	/	<0.01	5.0
	Wheat beer (b)	/	Water<br>Barley malt<br>Wheat<br>Hop<br>Coriander<br>Dried orange peals		/	/		/		/	/	/	/	/	/	<0.1	4.9
	Wheat beer (c)	/	Water<br>Barley malt<br>Wheat		/	/		/		/	/	/	/	/	/	<0.1	4.5
	Wheat beer (d)	/	Water<br>Barley malt<br>Wheat malt<br>Hop<br>Coriander<br>Orange peals		/	/		/		/	/	/	/	/	/	<0.1	4.7
	Ale (bottle fermented) (a)	/	Water<br>Barley malt<br>Hop<br>Yeast	 20	Sugar (refermentation)	/		/		/	/	<0.5 (0.0)	/	/	/	<0.01	7.8
	Ale (botle fermented) (b)	/	Water<br>Barley malt<br>Hop<br>Hop extract<br>Yeast		Glucose syrup<br>Sugar	/		/		/	57	0 (0)	4.0 (0.0)	/	0.4	<0.01	7.0
	Ale (bottle fermented) (c)	/	Water<br>Barley malt<br>Hop<br>Yeast		/	/		/		/	56	<0.5 (0.0)	/	/	/	<0.01	8.0
	Ale (bottle fermented) (d)	/	Water<br>Barley malt<br>Hop<br>Yeast		Sugar (refermentation)	/		/		/	52	<0.5 (0.0)	3.8 (0.0)	/	<0.5	<0.01	8.5
	Ale (bottle fermented) (e)	/	Water<br>Barley malt<br>Hop<br>Yeast		Sugar	/		/		/	58	<0.5 (0.0)	4.3 (<0.5)	/	0.5	<0.01	6.8

* Some beverages do contain ascorbic acid as an antioxidant, but if the exact dose per 100 mL is not mentioned on the label it is not included here, only in the main ingredients list
